# A Case of Asymptomatic Lead Poisoning in a Two-Year-Old With a Background of Pica and Iron Deficiency Anaemia

**DOI:** 10.7759/cureus.113253

**Published:** 2026-07-23

**Authors:** Rafia Ayub, Inayat K Hafiz, Ahsan Ul-Haq

**Affiliations:** 1 Internal Medicine, Manchester University NHS Foundation Trust, Manchester, GBR; 2 Paediatrics, Manchester University NHS Foundation Trust, Manchester, GBR

**Keywords:** iron deficiency anaemia (ida), lead poisoning, paediatric lead poisoning, pica, screening

## Abstract

Lead poisoning remains an important yet under-recognised cause of morbidity in children. We report the case of a two-year-old child presenting with asymptomatic lead poisoning identified following investigation of pica, including ingestion of wall paint. This was with concomitant iron deficiency anaemia secondary to excessive cow’s milk intake. Given these significant risk factors, blood lead level screening was done despite her asymptomatic presentation, revealing a markedly elevated lead level of 3.34 µmol/L (reference range ≤ 0.10 µmol/L). Following these results, she was admitted for treatment with chelation therapy under the guidance of the National Poisons Information Service (NPIS), with ongoing surveillance in clinic to monitor treatment response and ensure a decline in blood lead levels. This case highlights the diagnostic challenges of asymptomatic lead poisoning and emphasises the importance of having a low threshold for screening in children with risk factors, including pica, nutritional deficiencies, and potential environmental lead exposure. Early recognition can facilitate timely treatment, reducing the risk of long-term complications.

## Introduction

Lead poisoning is a toxic condition caused by lead accumulation in the body. Although environmental exposure has fallen following restrictions on the use of lead-containing materials, it remains an important, preventable cause of morbidity, particularly in children. Young children are especially vulnerable as they absorb up to four to five times more lead than adults from the same ingested dose [1]. Frequent age-appropriate hand-to-mouth and object-to-mouth behaviours, which start at around six months of age and roughly start to decline from ages three and upwards [2], further increase the risk of exposure to lead-containing materials. Additional risk factors include pica - the compulsive, habitual consumption of non-food items - and nutritional deficiencies, particularly iron deficiency, which increases gastrointestinal lead absorption.

It can be difficult to detect lead poisoning in children because it is frequently asymptomatic or presents with non-specific symptoms. As lead accumulates in multiple organs, particularly the brain and bones, the clinical manifestation of lead poisoning is varied; it ranges from gastrointestinal symptoms such as abdominal pain, vomiting and constipation, to neurological manifestations including behavioural change, distal neuropathies, and, in severe cases, encephalopathy [3].

There is no recognised safe blood lead concentration in children. Confirmation of cases requires venepuncture for whole blood levels. Evaluation should also increase screening for other nutritional deficiencies, particularly iron deficiency, and investigation of potential environmental sources of exposure. Plain radiographs may demonstrate metaphyseal ‘lead lines’ in children with significant exposure, suggestive of arrested bone growth [3].

Treatment involves identification and removal of the source of exposure and, in cases of significant toxicity, chelation therapy. Ongoing neurodevelopmental follow-up may be required as neurological injury may be irreversible despite treatment.

We report the case of a two-year-old child with asymptomatic lead poisoning identified through targeted blood lead screening prompted by pica and iron deficiency anaemia.

## Case presentation

A two-year-old girl was referred to the paediatric outpatient clinic in September 2025 following recent consumption of non-nutritive substances, or pica, including peeling paint from the walls. She had a known background of iron deficiency anaemia secondary to excessive cow’s milk intake, with no other identified underlying cause of anaemia, including inherited causes, such as thalassaemia. She was on regular Macrogol 350 (with potassium chloride, sodium bicarbonate, and sodium chloride; two sachets daily) to ensure opening of her bowels once daily. She was eating, but her appetite was slightly reduced.

The girl was born preterm at 33.5 weeks via a caesarean section for maternal pre-eclampsia. She required 23 days of neonatal care. Her developmental age was appropriate. Her siblings were fit and well, and there was no family history of similar complaints.

On examination, she appeared well, and full systemic examination was completely normal, other than a general appearance of pallor. However, given this pica background and known iron deficiency anaemia, the clinical suspicion for further nutritional deficiencies and potential heavy metal exposure was raised. She had bloods taken, including full blood count (FBC), liver function tests (LFTs), iron profile, lead levels, and vitamin D (see Table 1).

**Table 1:** Blood test results from the September 2025 outpatient clinic, demonstrating raised lead levels of 0.89 µmol/L.

Blood test	Normal range	Result from the September clinic visit
White blood cells	6.0-17.0 10^9^/L	7.3
Haemoglobin	101.0-138.0 g/L	52
Haematocrit	0.300-0.400 L/L	0.230
MCV (mean corpuscular volume)	73.00-88.00 fL	49.90
MCH (mean corpuscular haemoglobin)	24.0-30.0 pg	11.3
MCHC (mean corpuscular haemoglobin concentration)	310-350 g/L	226
Neutrophils	1.00-8.50 10^9^/L	2.41
Alanine transaminase (ALT)	1-35 IU/L	19
Alkaline phosphatase (ALP)	90-540 U/L	214
Total bilirubin	0-21 µmol/L	4
Albumin	28-40 g/L	30
Iron	5.0-25.0 µmol/L	1.4
Transferrin	2.20-3.37 g/L	4.06
Transferrin saturation	15.0-45.0 %	1.4
Lead	<0.1 µmol/L	0.89
Total 25-hydroxy vitamin D	>50 nmol/L	18.8

Given the low haemoglobin levels, she was asked to attend hospital. After discussion with haematology, they advised discharging with oral iron replacement to complete a three-month course.

The lead level was also raised at 0.89 µmol/L. Public health authorities were informed, and there was a plan put in place for her lead levels to be monitored regularly following these raised results. There was also discussion surrounding the environmental source for the lead ingestion, believed secondary to paint in the house, which the patient’s mother had been trying to reduce.

On outpatient follow-up in early January, the patient was clinically well. The mother reported that the patient’s appetite had been improving. Her systemic examination was once more normal, though there was still mild pallor. Bloods were repeated, including repeat lead levels, and she was sent home from clinic.

Then, at the end of January, her blood lead levels returned as 3.34 µmol/L (normal <0.1 µmol/L), indicating a significantly elevated result. The paediatric consultant discussed this result with the National Poisons Information Service (NPIS) regarding this result, and a plan was made for the child to be admitted to the Children’s Unit at the hospital for treatment. The treatment in question was chelation therapy with calcium disodium EDTA (ethylenediaminetetraacetic acid) at a dose of 75 mg/kg intravenously, with daily monitoring of bloods, including renal function, liver function, and lead levels. They also requested additional tests, including zinc levels (to be replaced if required), coeliac screen, and repeat vitamin D levels. The patient was to have their urine output monitored, with administration of intravenous fluids if necessary, and their clinical state monitored for any signs of encephalopathy. There was also a request for a plain radiograph of the long bones to assess growth plates and bone density. The patient’s parents were contacted, and she was brought to hospital.

As an inpatient, the patient remained clinically well on examination with good appetite. Their bowels habits remained normal. A more in-depth history surrounding the pica background was obtained, uncovering that the paint was peeling from the walls in a rented house, and it was unclear how long it had been since the paint was replaced.

As an inpatient, her admission bloods were as follows (see Table 2).

**Table 2:** Blood test results from January 2026 admission, demonstrating a lead level of 1.64 µmol/L.

Blood test	Normal range	Result on admission
White blood cells	6.0-17.0 10^9^/L	11.6
Haemoglobin	101.0-138.0 g/L	89
Haematocrit	0.300-0.400 L/L	0.340
MCV (mean corpuscular volume)	73.00-88.00 fL	56.10
MCH (mean corpuscular haemoglobin)	24.0-30.0 pg	14.7
MCHC (mean corpuscular haemoglobin concentration)	310-350 g/L	262
Neutrophils	1.00-8.50 10^9^/L	5.32
Sodium	133-146 mmol/L	139
Potassium	3.5-5.0 mmol/L	4.4
Urea	2.5-6.5 mmol/L	2.5
Creatinine	15-31 µmol/L	23
Alanine transaminase (ALT)	1-35 IU/L	16
Alkaline phosphatase (ALP)	90-540 U/L	245
Total bilirubin	0-21 µmol/L	3
Albumin	28-40 g/L	36
Adjusted calcium	2.20-2.70 mmol/L	2.47
Phosphate	1.10-2.00 mmol/L	1.50
Magnesium	0.70-1.00 mmol/L	0.83
Parathyroid hormone	1.2-6.3 pmol/L	1.8
Iron	5.0-25.0 µmol/L	2.0
Transferrin	2.20-3.37 g/L	3.96
Transferrin saturation	15.0-45.0%	2.0
Lead	<0.1 µmol/L	1.64
Zinc	10.0-18.0 µmol/L	7.7
Total 25-hydroxy vitamin D	>50 nmol/L	96.7
TTG (tissue transglutaminase) IgA antibody (serum)	0.0-14.9 kU/L	<0.5

**Figure 1:**
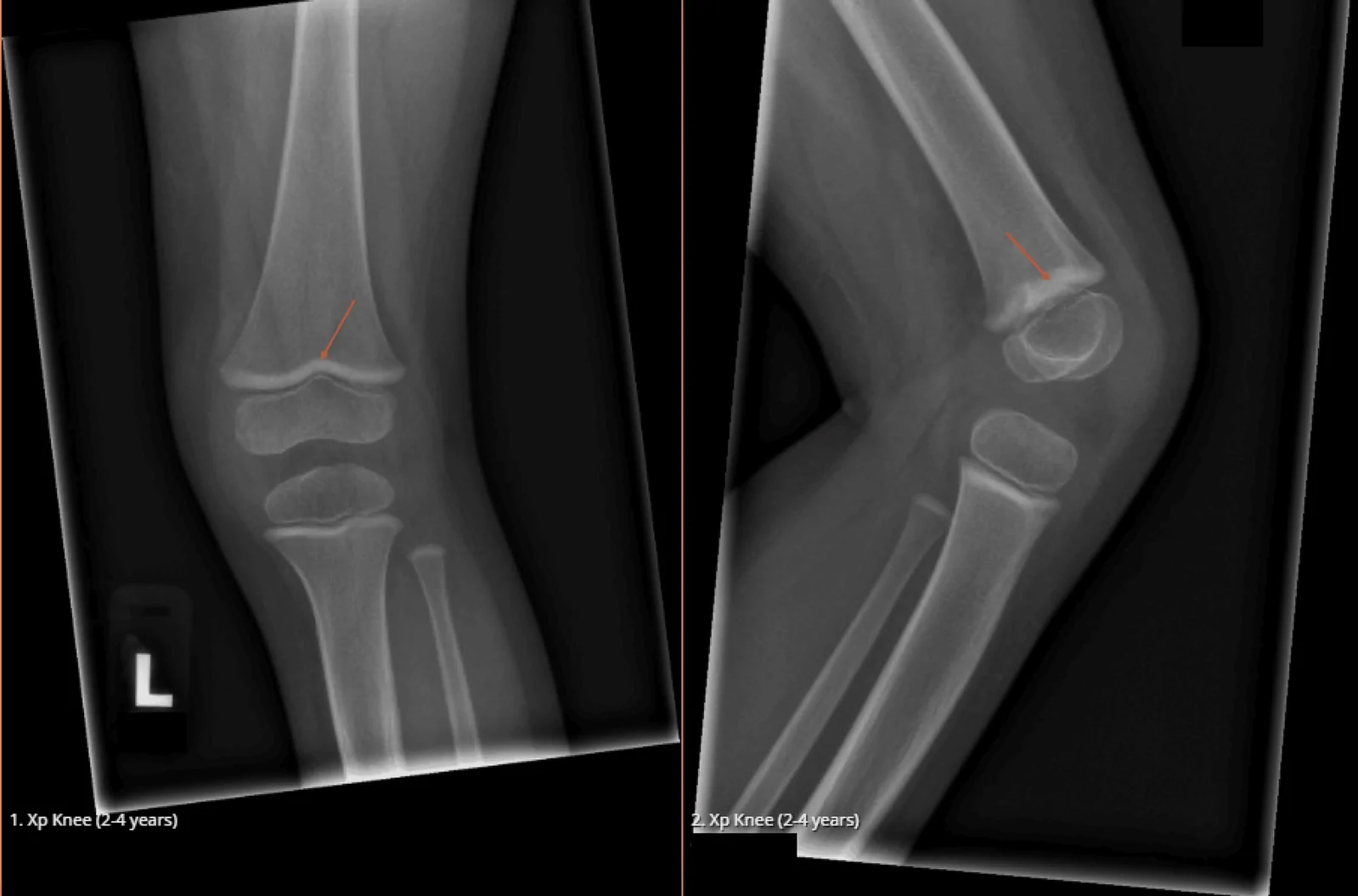
Plain radiograph of the left knee: anterior-posterior view on the left and lateral views on the right. There is mild increased metaphyseal sclerosis, but no further metaphyseal sclerotic bands. Bone density appears normal. 'L' = left; 'Xp' = X-ray photograph

She had a plain radiograph of her left knee including the distal femur and proximal tibia to assess for growth plate changes, which showed mildly increased metaphyseal sclerosis - a feature of lead poisoning (see Figure 1).

Her renal function remained stable as an inpatient. She received a total of five days of chelation therapy. Due to a turnover of seven days for lead levels to return, she was discharged without any confirmed lead levels, with a plan to repeat the levels after her discharge with regular follow-up.

Three months after this hospital stay, she attended a follow-up clinic, where she had repeat blood tests (see Table 3) and was clinically assessed. She had been doing well, and there had been no further exposure to paint following the installation of wallpaper in the home. Her dietary intake had improved, and she was no longer exhibiting pica behaviours. She was opening her bowels regularly, and her parents had no current concerns regarding constipation. Clinically, she appeared well, and her systemic examination was unremarkable.

**Table 3:** Blood test results from the April 2026 clinic, demonstrating a lead level of 1.60 µmol/L.

Blood test	Normal range	Result from the April clinic visit
Haemoglobin	101.0-138.0 g/L	96
MCV (mean corpuscular volume)	73.00-88.00 fL	55.30
Lead	<0.1 µmol/L	1.60
Iron	5.0-25.0 µmol/L	2.2
Transferrin	2.20-3.37 g/L	4.23
Transferrin saturation	15.0-45.0%	2.1

Her most recent lead levels taken on the last day of her January admission had measured 1.47 µmol/L, and although still elevated, displayed significant improvement compared to prior to admission. The lead levels taken in this clinic level were chased and returned as 1.60 µmol/L, showing a mild increase compared to previous (see Table 4 for the trend of lead levels). Given the rise, she is due for further follow-up with repeat bloods this July.

**Table 4:** Blood lead levels across the course of the clinical case.

Timeline	Stage in treatment	Lead levels in µmol/L
September 2025 clinic	Initial screening in the clinic	0.89
November 2025 clinic	Follow-up clinic lead levels prompting further monitoring	0.85
January 2026 clinic	Follow-up clinic lead levels prompting admission	3.34
Day 1 of January 2026 admission	Whilst on chelation therapy	1.64
Day 2 of January 2026 admission		1.56
Day 3 of January 2026 admission		1.47
April 2026 clinic	First follow-up post-chelation therapy	1.60

## Discussion

This case demonstrates that clinically significant lead poisoning may occur in completely asymptomatic children. Reliance on symptoms may delay diagnosis, allowing for progression of toxicity. In this case, the patient was identified before developing clinical manifestations, highlighting the value of screening based on risk factors rather than symptoms alone. In this patient, several recognised risk factors were present: pica, iron-deficiency anaemia, and developmentally appropriate hand-to-mouth behaviours. Together, these factors prompted blood lead level screening despite the absence of symptoms and ultimately led to early diagnosis. This is particularly important because there is no recognised safe blood lead concentration in children, and adverse neurodevelopmental effects have been demonstrated even at relatively low levels. Early identification before the onset of symptoms is thus essential to minimise the risk of irreversible neurodevelopmental injury.

Pica substantially increases the likelihood of ingesting lead-containing materials. Common sources include peeling paint from the walls, plaster, soil, and dust. Although lead-based household paint has been banned in the UK since 1992 [4], legacy paint remains an important source of childhood exposure. In this case, ingestion of paint was considered the most likely source of lead, although the precise age and origin of the paint could not be confirmed. Similar cases have been reported. For example, a 2016 case report [5] by Jouhadi et al described lead poisoning secondary to pica, reinforcing the importance of screening children with recognised risk factors.

The presence of iron deficiency anaemia in this case is also pertinent given the link between iron deficiency and lead absorption. As both metals share some transport pathways, absorption of lead is increased in iron deficiency, rendering these patients more susceptible to lead toxicity [6]. It is therefore vital that iron deficiency be corrected promptly, whether through oral or intravenous replacement; in this case, the patient started oral iron replacement early on in their clinical course. Iron deficiency is therefore an important modifiable risk factor for lead toxicity.

Chelation therapy utilised in this case was indicated due to the markedly elevated blood lead levels. Chelators work by forming tight chemical bonds with heavy metals, thereby enabling them to be excreted. Although effective in reducing circulating lead, it does not remove lead stored within bone, meaning blood lead concentrations may rebound following treatment. In this patient’s case, her levels stagnated at 1.60 μmol/L in her most recent follow-up (see Table 4), which may reflect redistribution of lead from skeletal stores following chelation therapy, although continued environmental exposure must be excluded. Ongoing surveillance of lead levels is therefore essential.

Perhaps the most important learning point from this case is that chelation alone is insufficient if the environmental source of exposure is not identified and eliminated. It is compulsory to report all cases of lead poisoning levels ≥ 0.24 μmol/L to relevant public health authorities, who then carry out investigations to identify the lead source [7]. Without these steps, blood lead levels may continue rising, and repeated chelation therapy may become necessary. In this case, although the paint seems the likely source of lead toxicity, it is only through thorough investigations and routine blood lead levels during follow-ups that we can assure the source has been identified and removed successfully, reducing the risk of ongoing or recurrent exposure.

This case highlights that targeted blood lead level screening in children with pica, iron-deficiency anaemia and other recognised risk factors can facilitate early diagnosis and treatment before the development of irreversible neurodevelopmental sequelae.

## Conclusions

This case highlights that significant lead poisoning may occur in young children despite the complete absence of clinical symptoms. The combination of pica, iron-deficiency anaemia, and potential exposure to deteriorating household paint should prompt clinicians to have a low threshold for measuring blood lead levels, even in otherwise well children. Early identification allowed timely initiation of chelation therapy and environmental intervention before the development of overt lead toxicity, reducing the risk of long-term neurodevelopmental sequelae. This case reinforces the importance of recognising risk factors, addressing nutritional deficiencies, investigating environmental sources of lead exposure, and ensuring close multidisciplinary collaboration between paediatricians, toxicology services and public health teams to optimise patient outcomes.
